# The Durability and Efficacy of Cryopreserved Human Umbilical Cord Tissue Allograft for the Supplementation of Cartilage Defects Associated with the Sacroiliac Joint: A Case Series

**DOI:** 10.3390/reports6010012

**Published:** 2023-03-02

**Authors:** Albert Lai, Jon Shou, Steve A. Traina, Tyler Barrett

**Affiliations:** 1 Desert Pain Specialists, 72780 Country Club Drive, #C-300, Rancho Mirage, CA 92270, USA; 2 Regenative Labs, 1700 W Main Street, Pensacola, FL 32502, USA; 3 Western Orthopedics, 1830 Franklin Street, 450, Denver, CO 80218, USA

**Keywords:** Wharton’s jelly, regenerative medicine, sacroiliac joint dysfunction, cartilage defects

## Abstract

This Institutional Review Board (IRB)-approved retrospective observational protocol aims to report the safety and efficacy of birth tissue allografts applied in 38 patients with treatment-resistant sacroiliac (SI) joint pain. The research methodology consisted of an observational recording of the Numeric Pain Rating Scale (NPRS) and the Western Ontario and McMaster Universities Arthritis Index (WOMAC), which measure pain, stiffness, and physical function. No adverse events or adverse reactions were observed in the 38 patients. Statistically significant improvements in NPRS and WOMAC scores of the affected SI joint were reported after 90 days. The observational data suggests that Wharton’s jelly allograft applications are safe, minimally invasive, and efficacious. They may present an alternative to surgery for patients who fail conservative and procedural management of pain originating from chondral cartilage degeneration of the SI joint.

## 1. Introduction

Pain originating from the sacroiliac joint often mimics pain resulting from other anatomically juxtaposed structures, such as hip pathology, lumbar disc pathology, muscular strains, and nerve root injuries. SI joint pain can be multifocal in pathologic origin. However, axial loading and forcible rotation over time that causes deterioration of the articular cartilage protecting the joint is frequently observed in patients with SI pain. The sacroiliac joint is the largest in the body, with a surface area approaching 17.5 cm [1]. The SI joint has two component auricular cartilage facets, the sacral and the iliac. With age, sheering forces, and repetitive trauma, both the sacral and iliac facet cartilage exhibit morphological changes consistent with inhomogeneous extracellular matrix, fissuring, superficial irregularity of the articular surface, and chondrocyte clustering [2]. These degenerative changes constitute structural defects of the articular cartilage and may be primary pain generators. SI joint-mediated pain affects roughly 15% of the United States population, resulting in approximately 12 million physician visits yearly [3,4]. Treatment for SI joint pain and loss of income results in over 60 billion dollars in US healthcare costs annually [5]. The sacroiliac joint’s primary purpose is to lend stability and provide movement in three axes. Degenerative changes to the SI joint cartilage and trauma limit movement on the x-axis [6]. The resultant pain, functional impairment, and reduced quality of life can be disabling if not treated. Long-term symptomatic improvement in SI joint-related pain is not consistently supported in the medical literature, nor are the clinical benefits of intra-articular corticosteroids. Further, recent studies published in the Journal of Radiology suggest corticosteroids may cause more long-term harm to joint health than benefit [7].

Corticosteroids (CS), one of the most common injection treatments, have anti-inflammatory and immunosuppressive effects, which can not only lead to the reduction of vascular permeability but can also lead to the inhibition of inflammatory mediators that play a crucial role in the facilitation of immune cell infiltration [8,9]. While the exact mechanisms of corticosteroids are still unknown, it is theorized that the alteration of neutrophils inhibits them from reaching the inflammatory site [10]. Because of these side effects, corticosteroids have been clinically demonstrated to potentially worsen preexisting musculoskeletal conditions [11]. Current research suggests that CS injections should be used sparingly [12]. A controlled study involving 19 patients who had received CS (triamcinolone hexacetonide 40 mg/joint) to the SI joint bilaterally had transitory improvements in pain, stiffness, and spinal mobility, notable at one to three months after the injections. Nevertheless, by month 6, all 19 patients’ symptoms had returned to baseline, reversing any benefit recorded early in the study [13].

For those patients that exhaust all conservative and procedural options, SI fusion is a last-resort surgical intervention for refractory, symptomatic SI joint dysfunction. The procedure involves fusing the SI joint unilaterally or bilaterally through percutaneous or open technique. One to three dowels may be drilled through the joint to stabilize it. Screw fixation is another option, though reserved typically for traumatic fracture-related stabilization of the joint [14]. Although this intervention can relieve the symptomatic SI joint, the risk of complications is extremely high. Sacral incompetence fractures, instability of the contralateral SI joint, and stress on the hip joint below and the lumbar spine above prove common complications. One study concluded that 75% of SI fusion patients experienced adjacent segment degeneration within five years post-surgery [15]. While the surgery is a definitive approach to correcting the translational and painful movement of the SI joint, it does not correct the original cause of the pain as it does not contribute to the repair or regeneration of the degenerated SI joint articular cartilage. With unilateral SI fusion surgery approaching $35,000 in total expense, the need to investigate nonsurgical, durable alternatives to cortisone injections for refractory SI joint pain are imperative [16,17].

Wharton’s jelly (WJ), first discovered by Thomas Wharton in 1656, is a structural scaffolding tissue found in the umbilical cord, encompassing the two arteries and one vein [18]. WJ protects the umbilical cord’s vessels from external forces and allows for umbilical arterial and venous blood flow [19]. WJ has been reported to contain collagen types I and III, growth factors including insulin-like growth factor binding proteins 1,2,3,4 and 6, transforming growth factor alpha, and platelet-derived growth factor AA [19]. WJ was found to contain hyaluronic acid and immunomodulatory cytokines, including interleukin 6 receptor, interleukin 16, interferon gamma, and the anti-inflammatory cytokines, tumor necrosis factor receptor superfamily member 1A and 1B and interleukin 1 receptor antagonist [19]. It also contains proteoglycans and glycosaminoglycans. WJ and load-bearing articular cartilage share homologous cartilage extracellular matrix components and qualitative and quantitative characteristics [20]. These analogous biomechanical qualities make WJ an ideal allograft to supplement structural tissue defects in the articular cartilage of patients with refractory sacroiliac joint pathology. Clinical applications of WJ as a tissue supplement are increasingly being reported in the medical literature with positive functional quality of life and pain outcomes [21,22,23]. The application of WJ for structural tissue defects in load-bearing articular cartilage centers on supplementing the body’s chondrogenic response to injury instead of the fibrin-based response to injury. WJ supplementation promotes collagen-based articular cartilage scaffolding instead of fibrin-based scar tissue formation.

Addressing symptomatic articular cartilage defects in patients with SI joint-related complaints remains a clinical challenge. Most clinicians agree that nonsurgical intervention is the standard of care, yet current literature has yet to prove a reasonable and durable procedural alternative to steroid injections.

## 2. Case Presentation Section

All methods complied with the FDA and American Association of Tissue Banks (AATB) standards. The study was conducted in accordance with the Declaration of Helsinki and approved by the Institutional Review Board of the Institute of Regenerative and Cellular Medicine (IRCM-2022-311) on 12 January 2022. Informed consent was obtained from all patients. All data were analyzed by percent change analysis, as well as analysis of variance (ANOVA).

### 2.1. Material Collection and Preperation

After written consent, perinatal birth tissues were obtained from mothers following full-term Caesarian section deliveries. Prior to delivery, perinatal birth tissue donors completed a comprehensive medical, social, and blood test screening process. An independent certified laboratory tested all donations for infectious disease in accordance with Clinical Laboratory Improvement Amendments (CLIA) of 1988, 42 CFR part 493, and FDA regulations (Appendix A). The lab followed ISO 7 Certified Lab procedures in the processing of Wharton’s Jelly sample processing. Wharton’s jelly samples were aseptically dissociated from the rinsed umbilical cord. After dissociation, 100 mg of Wharton’s jelly was suspended in approximately 2 mL of sterile Sodium Chloride 0.9% solution (normal saline). The cryoprotectant dimethyl sulfoxide (DMSO) was added, and the suspension was frozen at −40 °C for storage until application day. The sample was not combined with cells, tissues, or articles other than the exceptions outlined in 21 CFR Part 1271.10(a)(3) (Human Cells, Tissues, and Cellular and Tissue-Based Product Regulation).

### 2.2. Study Population

This observational data collection study analyzed 38 (18 males and 20 females) adult patients who met predetermined inclusion and exclusion criteria at 14 clinics throughout the United States. Eligible patients included adults older than 18 with an articular cartilage structural tissue defect within the sacroiliac joint. The mean age was 71 ± 6.8 years, with a range of 52–86 years old. In total, 17 patients received a Wharton’s Jelly allograft application to the right SI joint, and 21 received a Wharton’s Jelly allograft application to the left SI joint.

### 2.3. Allograft Application

Fourteen clinics obtained umbilical cord tissue allografts. The application of the allografts was performed in a private medical setting. All patients underwent a professional medical history and physical examination and had exhausted over eight weeks of conservative management. All patients had radiographic evidence of cartilage degeneration of the symptomatic sacroiliac joint. All patients had failed prior systemic treatments that may have included in full or part Nsaid’s, muscle relaxants, physical therapy, pain medications, and diagnostic SI joint injections. All patients were examined on the day of Wharton’s Jelly allograft application to confirm symptoms site of application, and informed consent was obtained. Under sterile technique and with Fluoroscopic guidance, 2cc of Wharton’s jelly flowable allograft, or 100 mg of Wharton’s Jelly, was applied into the inferior third of the Sacroiliac joint after negative aspiration for blood. All patients were monitored for 30 min post-procedure. No patients experienced post-procedure complications and were all discharged home in stable condition. 

### 2.4. Questionnaire Composition

**Figure 1:**
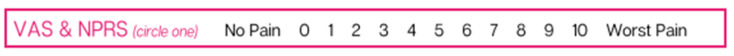
Numeric Pain Rating Scale used by patients to assess pain level.

**Figure 2:**
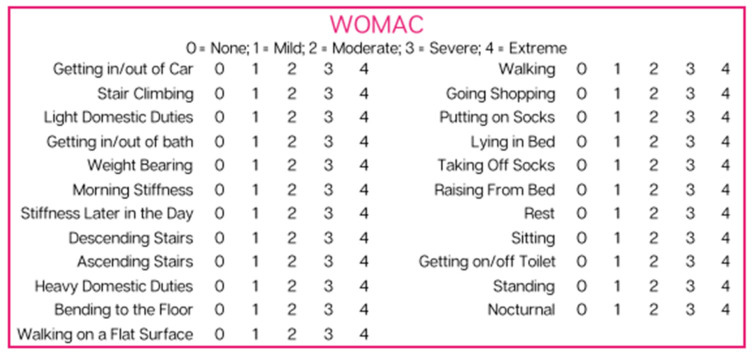
Western Ontario and McMaster Universities Arthritis Index used by patients to assess pain, stiffness, and function.

Patients were asked to fill out an initial questionnaire on the day of application consisting of NPRS and WOMAC scales (Figure 1 and Figure 2). Patients answered the same questionnaire 90 days after the initial allograft application. NPRS is a numerical pain scale employed as a subjective measurement of 0–10 for patients to rate their pain. A measurement of 0 indicates no pain, and 10 indicates the worst pain possible. WOMAC, measured on a scale of 0–4, is a combination of three questionnaires that measure pain, stiffness, and function. A measurement of 0 indicates that the patient is able to perform the activity with ease, and 4 indicates that the patient has extreme difficulty performing the activity. The total WOMAC score is calculated by adding each of the individual question scores together. The scores of these two scales were analyzed individually to allow for a more significant examination of the physical mobility of the affected joint. The reported scores for each patient can be found in Appendix B. 

## 3. Results

Changes were recorded from baseline data obtained on the day of application to 90 days following the application for both NPRS and WOMAC. At baseline, the average NPRS score was 6.7 ± 2.1. At 90 days, the average NPRS score was 3.6 ± 2.3. Thirty-two out of the 38 patients (84%) reported lowered NPRS scores. Most patients also reported an improvement in their WOMAC scores. At baseline, the average total WOMAC score was 56.1 ± 18.8. At 90 days, the average total WOMAC score was 43.3 ± 22.7. Twenty-nine (76%) reported lowered WOMAC scores at the 90-day mark. The average scores and percent change of NPRS and WOMAC scales are reported in Table 1.

The percent change analysis found that NPRS scores improved by an average of 42%, and WOMAC scores improved by an average of 22% at the 90-day mark. An analysis of variance for WOMAC scores revealed that the average final WOMAC score was statistically significant compared to the average initial WOMAC score, as evidenced by a *p*-value of 0.009, as seen in Table 2.

Initial and final NPRS and WOMAC scores were also analyzed by gender, as seen in Table 3.

The percent change analysis shows that while females reported higher initial and final NPRS and WOMAC scores, males showed a higher percentage of improvement. On average, females improved by 37% on the NPRS and 22.9% on the WOMAC. Males improved by 57% on the NPRS and 27.4% on the WOMAC.

The presented data displays the change from baseline data obtained on the day of application to the 90-day point following the application. These changes are clinically and statistically significant measures of improvement in patients with symptomatic cartilage degeneration in the treatment of refractory sacroiliac dysfunction.

## 4. Discussion

The presented observational data reports the results following the application of Wharton’s Jelly birth tissue allografts in 38 patients with chronic, symptomatic, treatment-resistant SI joint dysfunction. This IRB-approved study protocol observed that the WJ applications in all 38 patients were both safe and efficacious. Patients evaluated retrospectively demonstrated statistically significant improvements in pain, stiffness, and physical mobility of the SI joints. Statistically significant differences between baseline and 90-day reported scores in NPRS and WOMAC were observed. The results presented in this case provide a foundation for the future prospective study of Wharton’s jelly allografts as applied for articular cartilage defects contributing to treatment-resistant SI joint dysfunction.

The observed results from this observational case report align with 2022 data reporting the observational safety and efficacy of Wharton’s jelly as applied to cartilage defects associated with knee osteoarthritis [24]. The systemic review on allogenic umbilical cord tissue applied for articular cartilage degeneration of the knee was presented by Gupta in 2022. He concluded that Wharton’s Jelly should be considered in the armamentarium of clinicians dealing with middle-aged patients suffering from OA-related cartilage degeneration of the knee and are not surgical candidates yet [22]. To the best of our knowledge, this is the first observational data reporting the safety and efficacy of Wharton’s Jelly allograft applications to load bearding articular cartilage degeneration of the SI joint. In theory, all SI joint interventions serve to ameliorate a local, focal defect in the structural and supporting cartilage of the SI joint. Having failed attempts to allay symptoms from the systemic process that is Osteoarthritis, addressing the specific degeneration of the SI joint cartilage has been one of either temporary symptom reduction with steroid injections, joint denervation by radiofrequency ablation, and, more recently, viscosupplementation with hyaluronic acid. Should these procedures fail, Si joint fusion is the inevitable option to eliminate painful joint translation or dysfunction. However, SI joint fusion may accelerate load redistribution and increase adjacent segment disease of the lower lumbar spine or the hip’s juxtaposed acetabular joint.

In a 2015 study, Lindsey et al. demonstrated that fusion of the SI joint resulted in increased adjacent segment lumbar motion at L5-S1 above normal flexion, extension, lateral bending, and axial rotation [25]. In a retrospective database study published by Schoell et al. in 2016, 469 patients were evaluated for fusion-related complications [17]. Overall, the complication rate was 13.2% at 90 days and 16.4% at six months, with up to a 5% incidence of novel lumbar pathology noted following the procedure documented more commonly in men.

While this observational data reports the safety and efficacy of Wharton’s jelly allografts as they are applied for structural tissue defects of the SI joint articular cartilage, it does have limitations. The study design was unblinded and observational. However, the primary outcomes of NPRS and WOMAC were patient-reported, eliminating the influence of an unblinded investigator. In addition, the potential for more statistically significant functional and pain improvements may have been noted if patients were offered a subsequent allograft application. Patients in this observational data repository were restricted to only one WJ allograft application. Future blinded, randomized, prospective studies, with serial applications of WJ allografts and follow-up over 6 and 12 months, may demonstrate more significant statistical improvement in NPRS and WOMAC as well as determine the overall durability of WJ allograft applications should a comparative study be performed with cortisone injections. A 1,3, and five-year longitudinal design might allow for a comparative analysis of patients that ultimately required SI fusion between those treated with conventional interventions and those who received WJ allograft applications. 

## 5. Conclusions

In conclusion, this observational data collected on 38 patients with treatment-resistant pain and dysfunction of the SI joint reports that Wharton’s Jelly allografts demonstrated clinically and statistically significant improvements in function, joint mobility, and pain. No adverse events were reported in the observed WJ allograft application patient group. Based on the overall incidence of SI joint dysfunction, new literature cautioning the use of serial intra-articular cortisone injections, and the increased reports of SI fusion post-operative complications, alternatives to the current standard of care are warranted. Future randomized and prospective studies may further substantiate the efficacy and durability of WJ perinatal tissue allografts compared to cortisone injections, viscosupplementation, and, ultimately, SI joint fusion.

## Figures and Tables

**Table 1:** Percent change analysis for initial and final NPRS and WOMAC scores.

Scale	Average Initial Score	Average Final Score	Percent Change
NPRS	6.7	3.6	42% decrease
WOMAC	56.1	43.3	22% decrease

**Table 2:** Analysis of Variance for WOMAC scores.

ANOVA						
***Source of Variation***	***Sum of Squares***	***df***	***Mean Square***	***F-value***	***p-value***	
WOMAC	3069.59210	1	3069.59	7.06082	0.00964	significant

**Table 3:** Percent change analysis for females and males based on initial and final NPRS and WOMAC scores.

Gender	Avg. Initial NPRS	Avg. Final NPRS	% Change NPRS	Avg. Initial WOMAC	Avg. Final WOMAC	% Change WOMAC
Females	6.9	4.3	37% decrease	66.4	51.2	22.9% decrease
Males	6.3	2.7	57% decrease	44.1	32	27.4% decrease

## Data Availability

Data can be found in Appendix A and Appendix B.

## Appendix A. Test Kits

1. HBcAb: Catalog number: 06P06, Abbott Laboratories, Abbott Park, IL, USA;
2. HbsAg: Catalog number: 06P02, Abbott Laboratories, Abbott Park, IL, USA;
3. HCV: Catalog number: 06P04, Abbott Laboratories, Abbott Park, IL, USA;
4. HIV1, HIV2, plus O: Catalog number: 06P01, Abbott Laboratories, Abbott Park, IL, USA;
5. HTLV-I/II: Catalog number: 06P07, Abbott Laboratories, Abbott Park, IL, USA;
6. RPR: Catalog number: 900025, Arlington Scientific, Springville, UT, USA;
7. HIV1, HCV, HBV, NAT: Catalog number: 303330, 303331, 303719, 303334, 303344;
8. WNV: Catalog number: 07001061190, Roche Diagnostics, Indianapolis, IN, USA.

## Appendix B. Patient Reported Scores

	**Gender**	**Age**	**Application Site**	**Initial NPRS**	**Initial Total WOMAC**	**90-Day NPRS**	**90-Day Total WOMAC**
Patient 1	M	70	R SI	7	59	1	7
Patient 2	F	71	R SI	3	43	4	23
Patient 3	F	71	L SI	4	44	4	23
Patient 4	F	73	R SI	6	52	2	41
Patient 5	M	76	L SI	5	39	2	30
Patient 6	F	72	L SI	8	69	2	38
Patient 7	M	74	L SI	4	47	1	32
Patient 8	M	72	R SI	9	30	2	30
Patient 9	F	65	R SI	9	85	5	84
Patient 10	F	52	L SI	8	68	4	66
Patient 11	F	65	L SI	9	83	4	81
Patient 12	M	72	L SI	9	30	3	30
Patient 13	F	52	R SI	8	68	5	68
Patient 14	M	76	R SI	2	19	1	12
Patient 15	F	78	L SI	8	75	2	23
Patient 16	M	66	L SI	6	36	4	23
Patient 17	M	71	L SI	8	64	4	40
Patient 18	M	76	L SI	4	46	3	44
Patient 19	M	77	L SI	6	46	3	40
Patient 20	F	76	L SI	6	54	9	68
Patient 21	F	74	L SI	5	39	4	28
Patient 22	M	65	R SI	6	71	4	45
Patient 23	M	71	R SI	10	28	4	44
Patient 24	M	75	R SI	10	36	1	31
Patient 25	M	69	R SI	5	45	3	46
Patient 26	F	77	L SI	6	65	4	35
Patient 27	F	74	L SI	7	61	4	65
Patient 28	F	70	L SI	7	66	8	74
Patient 29	F	65	R SI	5	84	4	72
Patient 30	M	72	R SI	2	30	0	2
Patient 31	M	65	R SI	7	60	0	18
Patient 32	F	77	R SI	9	92	10	86
Patient 33	F	79	L SI	10	92	9	78
Patient 34	F	84	L SI	8	73	3	12
Patient 35	F	72	R SI	5	64	3	46
Patient 36	M	69	R SI	6	63	5	60
Patient 37	M	75	L SI	8	45	8	43
Patient 38	F	86	L SI	8	59	4	59
